# *PICAFlow*: a complete R workflow dedicated to flow/mass cytometry data, from pre-processing to deep and comprehensive analysis

**DOI:** 10.1093/bioadv/vbad177

**Published:** 2023-12-04

**Authors:** Paul Régnier, Cindy Marques, David Saadoun

**Affiliations:** Immunology-Immunopathology-Immunotherapy (i3) Laboratory, INSERM UMR-S 959, Sorbonne Université, 75005 Paris, France; Biotherapy Unit (CIC-BTi), Inflammation-Immunopathology-Biotherapy Department (DHU i2B), Groupe Hospitalier Pitié-Salpêtrière, Assistance Publique-Hôpitaux de Paris (AP-HP), 75013 Paris, France; Immunology-Immunopathology-Immunotherapy (i3) Laboratory, INSERM UMR-S 959, Sorbonne Université, 75005 Paris, France; Biotherapy Unit (CIC-BTi), Inflammation-Immunopathology-Biotherapy Department (DHU i2B), Groupe Hospitalier Pitié-Salpêtrière, Assistance Publique-Hôpitaux de Paris (AP-HP), 75013 Paris, France; Département de Médecine Interne et Immunologie Clinique, Sorbonne Université, AP-HP, Groupe Hospitalier Pitié-Salpêtrière, 75013 Paris, France; Centre National de Référence Maladies Autoimmunes Systémiques Rares, Centre National de Référence Maladies Autoinflammatoires et Amylose Inflammatoire, Inflammation-Immunopathology-Biotherapy Department (DMU 3iD), 75013 Paris, France; Immunology-Immunopathology-Immunotherapy (i3) Laboratory, INSERM UMR-S 959, Sorbonne Université, 75005 Paris, France; Biotherapy Unit (CIC-BTi), Inflammation-Immunopathology-Biotherapy Department (DHU i2B), Groupe Hospitalier Pitié-Salpêtrière, Assistance Publique-Hôpitaux de Paris (AP-HP), 75013 Paris, France; Département de Médecine Interne et Immunologie Clinique, Sorbonne Université, AP-HP, Groupe Hospitalier Pitié-Salpêtrière, 75013 Paris, France; Centre National de Référence Maladies Autoimmunes Systémiques Rares, Centre National de Référence Maladies Autoinflammatoires et Amylose Inflammatoire, Inflammation-Immunopathology-Biotherapy Department (DMU 3iD), 75013 Paris, France

## Abstract

**Summary:**

*PICAFlow* is a R-written integrative workflow dedicated to flow/mass cytometry data handling, from pre-processing to deep and comprehensive analysis. It is designed as a powerful all-in-one tool which contains all the necessary functions and packages presented in a user-friendly and ease-to-use fashion. *PICAFlow* also includes important features that are very frequently lacking in other close software, such as interactive R Shiny applications for real-time data transformation and compensation as well as normalization methods aiming to remove batch effects and unwanted inter- and intra-group heterogeneity. It also allows to perform dimensionality reduction, cell clustering (using different available approaches), as well as complementary statistical analyses and export different support for data interpretation and visualization.

**Availability:**

*PICAFlow* is available as a R-written package hosted at the following GitHub repository: https://github.com/PaulRegnier/PICAFlow and is complemented by a fully detailed tutorial available at the following URL: https://paul-regnier.fr/tutoriel-picaflow/.

## 1 Introduction

Since the early development of flow cytometry devices in the late 1960s (Dittrich and Göhde 1969), exponential progresses were made in this field. Nowadays, derived technologies like mass cytometry (CyTOF) (Zhang *et al.* 2023) and spectral cytometry (Ferrer-Font *et al.* 2020) allow to record at the single-cell level about 50 parameters (and even more) in one experiment. This evolution led to the constant increase of dataset size and complexity, both in terms of number of samples and parameters. Over the last decade, a growing number of R-written bioinformatic tools were developed to handle and analyze these data such as *flowCore* (Hahne *et al.* 2009), *SPADE* (Qiu *et al.* 2011), *openCyto* (Finak *et al.* 2014), *FlowSOM* (Van Gassen *et al.* 2015), *PhenoGraph* (Levine *et al.* 2015), *Cytofkit* (Chen *et al.* 2016), *SPADEVizR* (Gautreau *et al.* 2016), *CyTOF* (Nowicka *et al.* 2017), *ggCyto* (Van *et al.* 2018), *CytoML* (Finak *et al.* 2018), *CytoNorm* (Van Gassen *et al.* 2020), *CytoTree* (Dai *et al.* 2021), *CytofIn* (Lo *et al.* 2022), *cyCombine* (Pedersen *et al.* 2022), *flowStats*, etc. Astonishingly, very few include all the necessary functions to handle raw data and to perform pre-processing steps as well as deep and thorough analyses leading to simple and interpretable outputs and results. Here, we present *PICAFlow*, an all-in-one integrated R workflow which is dedicated to raw cytometry data pre-processing and deep analysis, in a user-friendly and easy-to-use fashion. This pipeline includes a lot of useful functionalities, such as parameters renaming and subsetting, compensation (only relevant for flow cytometry data), transformation, normalization, gating, downsampling, dataset splitting, dimensionality reduction, cell clustering, and production of statistical and graphical elements to interpret the data as deeply and thoroughly as possible.

## 2 General overview of *PICAFlow*

**Figure 1.**
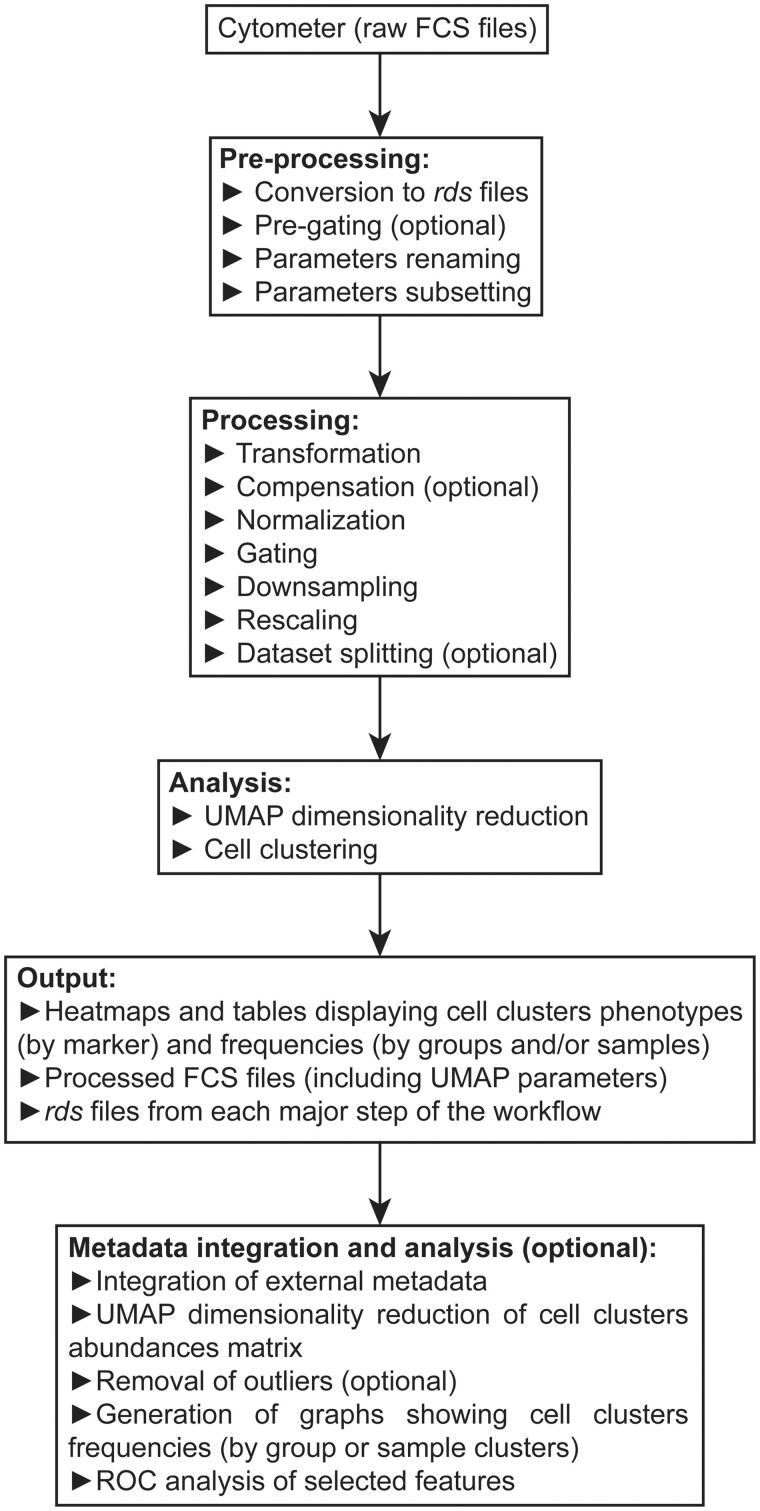
Overview of the *PICAFlow* workflow. UML diagram describing the whole *PICAFlow* workflow. *PICAFlow* can fully process flow/mass cytometry data from raw FCS files to identification of features of interest, for instance cell populations specific of a given group of interest, both at the phenotype and abundance levels.

*PICAFlow* is thought to be used as an all-in-one tool, thus providing selected functionalities coming from different R packages (which are listed in Section 3). The goal of *PICAFlow* is to completely process the dataset, from raw FCS files opening to generation of visualization graphs and tables, without the need to master other tools. In substance, *PICAFlow* could be seen as a wrapper of the most efficient parts of other R packages, encapsulated as user-friendly and easy-to-use functions. Figure 1 shows the actual workflow performed by *PICAFlow*. Among its main advantages compared to other solutions such as *Cytofkit* (Chen *et al.* 2016) or *CyTOF* (Nowicka *et al.* 2017) are its simplicity of use and its separation into easily comprehensive and complementary steps. *PICAFlow* was designed around two main principles: (i) to be usable by users with no to low bioinformatics and programming background, and (ii) to provide all the necessary tools to fully analyze datasets and extract as much biologically relevant information as possible from them.

## 3 Packages used by *PICAFlow*

*PICAFlow* uses a vast collection of R packages to perform its routine, which are detailed as follow. Parallelized operations and optimized loops use *parallel*, *doSNOW*, and *foreach*. Cytometry-related data handling uses *flowCore*, *flowStats*, *flowWorkspace*, and *flowGate*. Plots and heatmaps generation uses *ggplot2*, *ggcyto*, *gplots*, *plotly*, and *cowplot*. Dimensionality reduction through Uniform Manifold Approximation and Projection (UMAP) computing uses *uwot*. Receiver operating characteristic (ROC) analyses use *ROCit*. Text-based data import uses *utils* and *readxl*. Addition of interactivity to data transformation uses *shiny*. Exceptions capture uses *attempt*. Density peaks identification uses *pracma*. Cell clustering uses *class*, *FlowSOM*, and *FastPG*. Progress bars use *tcltk*. Miscellaneous operations use *Biobase*, *matrixStats*, *methods*, *rlang*, and *stats*.

## 4 Pre-processing of raw FCS files

To allow a relevant future analysis of data, raw FCS files must be pre-processed following several important steps. The first one, only relevant for flow cytometry data, is typically incarnated by the pre-gating of cells of the desired shape and/or single cells thanks to SSC/FSC and derived parameters, which is actually made possible in *PICAFlow* thanks to a dedicated R Shiny application we developed. Afterwards, users should subset the embedded parameters to only extract the ones that will be used for the subsequent analyses. Typically, time-based and SSC/FSC parameters (only relevant in flow cytometry data) are removed at this step, while fluorescence- or metal-based channels are conserved. In the same time, the remaining parameter names can be changed to more user-friendly values. Throughout these steps, data are handled and then finally exported as *rds*-formatted files to ease the computing of the next steps.

## 5 Transformation

Next, data are transformed using any function among the ones currently implemented in *PICAFlow*: *logicle*, *biexponential*, or *arcsinh*. *PICAFlow* includes a R Shiny-based interactive application to observe and manually tune in real-time the associated parameters (*t*, *w*, *m*, and *a* for *logicle*, *a*, *b*, *c*, *d*, *f*, and *w* for *biexponential* and *a*, *b*, and *c* for *arcsinh*, as defined by the respective functions from the *flowCore* package). In the case of the *logicle* transformation, the interactive application also allows to automatically determine them. When completed, each parameter value for each cytometry channel is exported to a summary *rds* file and then applied to each sample. Here, the main advantage of *PICAFlow*, as compared to other all-in-one workflows such as *Cytofkit* (Chen *et al.* 2016) or *CyTOF* (Nowicka *et al.* 2017), is to provide an intelligible, visualizable, and interactive way to transform the data in real-time, which is, to our knowledge, something that is only proposed by commercial software such as FlowJo or Kaluza. Furthermore, most packages allowing the user to transform cytometry data, even if they let users fully control the used parameters, never provide them a way to observe in real-time what they are actually doing to the data. More importantly, most transformation functions were originally defined in their respective packages using fixed/hard-coded values for their parameters, which are very often not optimal or even totally irrelevant as the cytometry technology as a whole evolved. Consequently, we strongly believe that our interactive R Shiny application will dramatically help users to correctly transform their data, thus avoiding new mathematical biases introduction, potentially leading to incorrect downstream interpretation.

## 6 Compensation

Next, the compensation matrix should also be thoroughly checked and eventually tuned if needed. This step is generally not relevant for mass cytometry data, but can still be performed if desired. Hopefully, *PICAFlow* also includes a dedicated R Shiny application to perform this step in a visual and interactive fashion. When the compensation matrix is considered as optimal, users can apply it to the dataset. On the other hand, users that do not want to tune the compensation matrix can directly apply sample-wise the compensation matrices that are self-contained within each sample. Even if the pre-gating and/or compensation tuning steps could be done, if desired, within classical flow/mass cytometry acquisition and/or analysis software such as BD FACSDiva (BD Biosciences), FlowJo (BD Life Sciences), CytExpert (Beckman Coulter), CyTOF (Fluidigm), Kaluza (Beckman Coulter), or FCS Express (De Novo Software), *PICAFlow* allows users to perform them directly in R. In our opinion, this represents one important strength of *PICAFlow*, because it can be used totally independently of conventional third-party analysis software. Noteworthily, these pre-gating and compensation tuning steps were intentionally made optional, because if they are needed, users still have the possibility to perform them in their favorite analysis software and export new FCS files that could also be used as input for *PICAFlow*.

## 7 Normalization

*PICAFlow* also includes the possibility to normalize the dataset of interest, in order to correct for batch effects as well as unwanted inter- and intra-group heterogeneity. In this step, peaks for each cytometry channel and sample are determined using a modified version of *gaussNorm* method (adapted from *flowStats* R package). These peaks can be manually adjusted if needed in the case the automatic determination fails or is not precise enough. Once completed, data are normalized channel-wise and graphical representation of signals before and after normalization are exported to visualize the efficiency of the process and correct the peak values if needed. Basically, the normalization helps to “realign” the peaks according to their relative position. For instance, if a given channel shows a bimodal distribution (typically “low” and “high” expressing cells), then the “low” and “high” peaks for each sample will first be determined, then all the “low” peaks will be aligned together, as well as the “high” peaks. This specific and flexible implementation of peaks edition in *PICAFlow* can be advantageously mixed with the more and more current flow/mass cytometry-associated good practice to use the same control sample in each batch, in order to better evaluate, and correct as well, the newly introduced batch effects. Interestingly, within the last years, other R packages dedicated to cytometry data normalization and/or batch-effect correction appeared, such as *CytoNorm* (Van Gassen *et al.* 2020), *CyCombine* (Pedersen *et al.* 2022), and *CytofIn* (Lo *et al.* 2022). Even if they offer different promising and robust approaches to normalize cytometry data, we unfortunately did not manage at the moment to correctly make them work in concert with *PICAFlow*. This is mainly because of huge discrepancies between our respective workflow designs and ways of handling data. Despite the existence of other normalization-focused methods, to our knowledge, *PICAFlow* is the only all-in-one cytometry data processing and analysis R package which also features a way to normalize data.

## 8 Gating

Afterwards, *PICAFlow* can proceed with the cells gating, using any available channel. We offer users the opportunity to interactively draw their gates through the use of a R Shiny application from the *flowGate* package. Gates of different shapes (rectangular, polygonal, quadrant, and 1D) can be precisely defined and positioned on 1D/2D histograms/dot plots. As, at this step, signals are normalized, one gate should fit all samples. Nonetheless, the gating process we implemented in *PICAFlow* still allows users to change the gates on some particular samples which were not perfectly gated by the previously drawn global gate. Interestingly, a whole gating strategy is even possible, by applying several gating processes iteratively, depending on the cell subpopulation of interest.

## 9 Downsampling and rescaling

Then, data are downsampled to generate a subdataset where all the groups contribute equally to the dataset, and where all the samples of a given group contribute equally to this group. Please note that this process is not destructive, because cells are simply “tagged” as being part of the downsampled dataset or not. During this process, users have the possibility to rescale the dataset, in a parameter-wise fashion. This was implemented to prevent the highly expressed parameters to “overwhelm” the lowly expressed ones just because of their absolute magnitude. Of note, the rescaling is applied on the whole dataset, without considering if a cell belongs to the downsampled dataset or not. This downsampled dataset will be used in the subsequent steps of the workflow (and notably in the dimensionality reduction and the cell clustering ones).

## 10 Dataset splitting (optional)

If needed, the dataset can be further split into two subdatasets, in a cell-wise fashion. This means that the cells of each sample are split, but not the samples themselves. In consequence, all the samples of the original dataset will be found in the two split datasets. This feature is typically interesting when one wants to construct “training” and “validation” datasets in the case the original groups do not contain enough samples to ensure a satisfying per-sample separation.

## 11 UMAP dimensionality reduction and cell clustering

**Figure 2.**
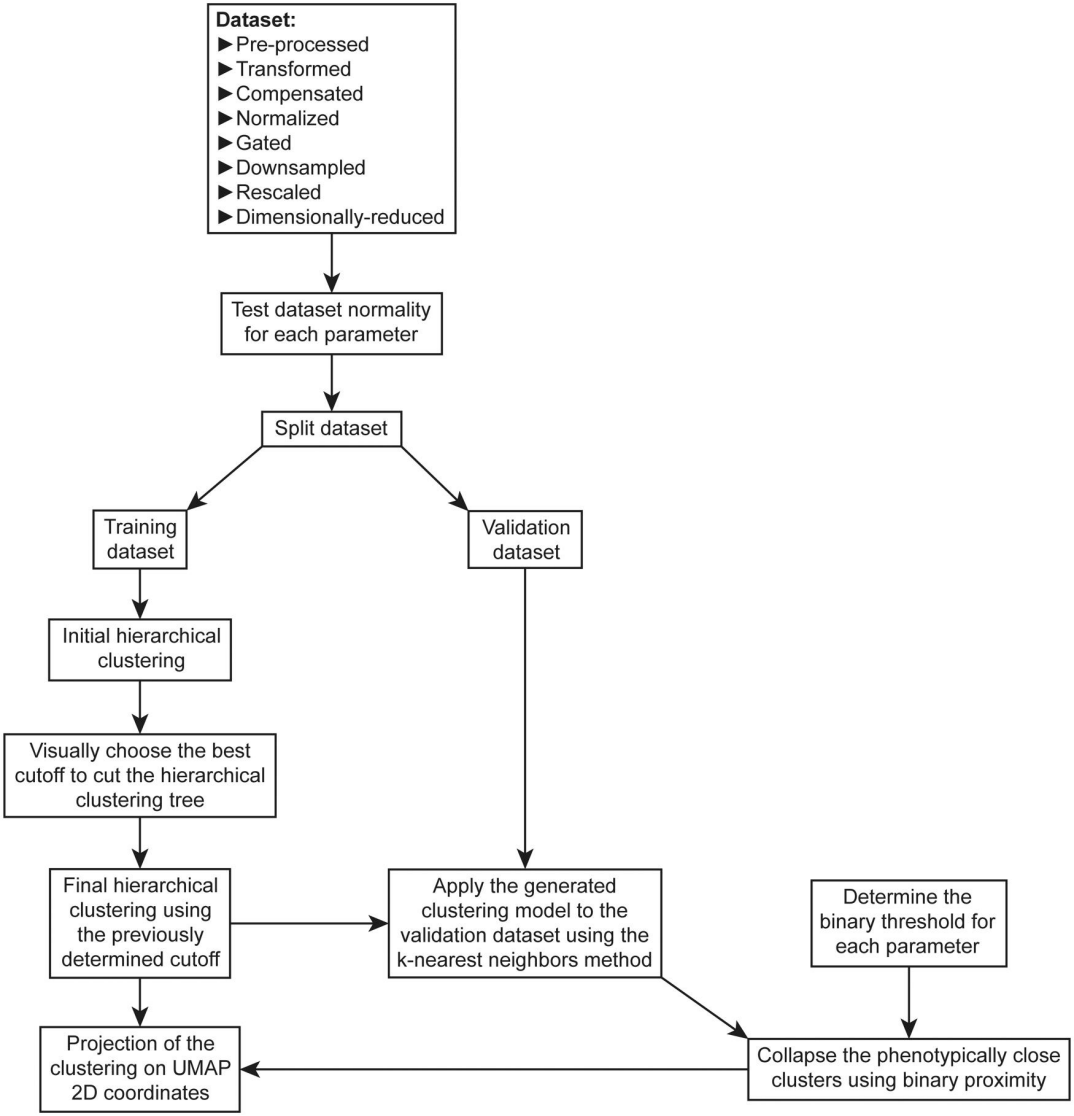
Overview of the new clustering method embedded within the *PICAFlow* workflow. UML diagram detailing the steps used for the new cell clustering method we incorporated in *PICAFlow*. This method involves the generation of a hierarchical clustering model on a “training” subdataset derived from the whole dataset of interest. Then, this method applies the clustering model to the remaining cells using the k-nearest neighbors approach. Finally, clusters are collapsed according to their binary proximity, based on the visually determined binary thresholds for each cytometry parameter.

Thereafter, dimensionality reduction analysis is performed using UMAP algorithm (McInnes *et al.* 2020) in order to create an overview 2D map of the dataset. We chose to implement UMAP because of its versatility and its superiority on a lot of different data types, including bulk transcriptomics (Yang *et al.* 2021) and single-cell RNA-Seq (Sun *et al.* 2019). Very few reports actually thoroughly investigated dimensionality reduction methods for cytometry data, but a recently published one highlighted that many, including UMAP, could honorably work and even be complementary, depending on the cytometry dataset used (Wang *et al.* 2023). After the dimensionality reduction step, users have the possibility to perform cell clustering using any implemented method within *PICAFlow*. We propose our own “guided semi-supervised” clustering method, fully detailed in Fig. 2, which is composed of several steps: first, a hierarchical clustering of a “training” subset of cells is performed, then the underlying model is generated and applied to the remaining “validation” cells using the k-nearest neighbors method. Finally, the number of clusters is reduced to a reasonable number (typically between 10 and 40) using the binary proximity of the obtained phenotypes, which is notably based on the visual determination of thresholds for each parameter negativity/positivity. At the end, the identified clusters are overlaid to the UMAP graph to ease their visualization and to look at their integrity and homogeneity. We also provide in the Supplementary Fig. S1 and in the Supplementary Material a comparison of our clustering method with two other already well-established methods: *FlowSOM* (Van Gassen *et al.* 2015) and *PhenoGraph* (Levine *et al.* 2015).

## 12 Output and visualization

At the end of the process, several outputs are produced to ease data interpretation, such as heatmaps and tables displaying the cell cluster phenotypes (by marker) and frequencies (by group and/or by sample), but also processed FCS files (including UMAP parameters) for further manual analysis and *rds* files containing data from each major step of the workflow.

## 13 Metadata integration and analysis

Finally, if needed, metadata of involved samples can be integrated to the dataset analysis, being for instance patient-associated clinical data. Then, UMAP dimensionality reduction is performed on the table containing all the clusters abundances for each sample. The generated 2D plot can thus be used to identify clusters of samples directly on the UMAP plot, but also to identify and eventually remove outliers (in this latter case, please proceed only if truly needed: please see the tutorial for more information). Furthermore, graphs showing cell cluster frequencies (either by groups or sample clusters) are generated, which can greatly help to isolate features that are specific to a given group (such as a disease, a specific condition, etc.). Ultimately, ROC analyses can also be performed to evaluate the specificity and sensitivity of selected features (cell populations, manually computed scores, etc.) in groups separation.

## 14 Application to real datasets

Even if it had no formal name at the time the following experiments were analyzed, we already successfully applied the *PICAFlow* methodology to a flow cytometry dataset consisting of peripheral blood mononuclear cells (PBMCs) from either patients suffering from Giant Cell Arteritis (GCA) or healthy donors (HD)(Régnier *et al.* 2023). GCA is one of the most common large vessel vasculitis in humans which can lead to serious complications due to aorta and main arteries inflammation and infiltration with immune cells. We reported in this paper that the CTLA-4 immune checkpoint was specifically and significantly upregulated both in blood and aorta of GCA as compared to controls. Using *PICAFlow*, we were able to handle, process and normalize our FCS files, before gating on CD4^+^ regulatory T cells (Tregs) which are characterized by a CD3^+^ CD4^+^ FoxP3^+^ CD25^hi^ phenotype. Then, we performed UMAP dimensionality reduction analysis as well as cell clustering using our approach which combines hierarchical clustering and k-nearest neighbors methods. We found two clusters of Tregs of precedingly undocumented phenotypes which showed radically and significantly different abundances in GCA as compared to controls. We notably showed that Tregs in GCA patients (i) were less abundant, (ii) were mainly poorly activated/suppressive, and (iii) greatly upregulated intracellular CTLA-4 expression as compared to Tregs from HD. Of note, these results were totally in line with recent literature about these cells in the disease.

We also applied the methodology described in *PICAFlow* in another dataset we generated, which involved more than 200 PBMCs samples from patients suffering from primary Sjögren’s syndrome, one of the most frequent chronic autoinflammatory/autoimmune disease. In addition to the clinical manifestations this disease implies (dry eyes and mouth, fatigue, muscles and joints pain as well as lungs, peripheral nerves or kidney involvement, etc.), patients also have a 15–20 times higher risk to develop lymphoma. To date, there are no biomarkers which could allow to precisely identify the high-risk patients and thus follow them more closely. *PICAFlow* helped us to identify circulating T and B cells subpopulations which are associated with lymphoproliferation and lymphoma and show a higher sensitivity and specificity to predict lymphoma than the existing clinical and biological items. At the date of *PICAFlow* release, this manuscript is still under finalization but should be submitted soon.

## 15 Conclusion

*PICAFlow* allows to completely process and analyze flow/mass cytometry data and greatly helps to extract informative features that specifically define groups or samples of interest. Its versatility, compatibility and simplicity of use makes *PICAFlow* a powerful tool to handle large datasets with great numbers of parameters and/or cells. *PICAFlow* also features very useful functions which are almost never implemented in similar workflows, such as R Shiny interactive applications for real-time data transformation and compensation as well as data normalization methods. Of course, a complete tutorial which includes full example code and even a test dataset is also available at https://paul-regnier.fr/tutoriel-picaflow/.

## Supplementary Material

vbad177_Supplementary_Data
