# Supplementary material for: PICAFlow: a complete R workflow dedicated to flow/mass cytometry data, from pre-processing to deep and comprehensive analysis
Source: Bioinform Adv. 2023 Dec 4;3(1):vbad177. doi: 10.1093/bioadv/vbad177 (PMC10713114; doi:10.1093/bioadv/vbad177)
Supplement: vbad177_Supplementary_Data [file vbad177_supplementary_data.docx]

**Supplemental Material**

**Comparison of *PICAFlow* cell clustering method to other already well-established ones**

We indeed used the same normalized dataset to assess their performance. We observe in **Figure S1A** that the three tested clustering methods give a relatively comparable number of final clusters (16 for ours, 20 for *FlowSOM* and 22 for *PhenoGraph*). When comparing the phenotypes of the clusters identified with all methods in one common heatmap, we observe that our method is able to identify cell populations that were already seen by the two others (**Figure S1B**). To ease further interpretation of these data, we determined meta-clusters of relatively homogeneous cell populations using the generated top dendrogram (split using red squares).

For instance, the far-right meta-cluster M21 is composed of 6 cell populations (*PhenoGraph_C4*, *PICAFlow_C391*, *PICAFlow_C386*, *PICAFlow_C389*, *FlowSOM_C10* and *PhenoGraph_C2*), three of which were generated using our approach. These resemble populations identified by other methods: *PICAFlow_C391* is rather close to *PhenoGraph_C4*, whereas *PICAFlow_C386* and *PICAFlow_C389* more look like *FlowSOM_C10* and *PhenoGraph_C2*. Interestingly, in this meta-cluster, where *FlowSOM* only identified 1 cluster, *PhenoGraph* and *PICAFlow* identified 2 and 3 distinct clusters, respectively.

Overall, meta-clusters where each clustering method identified at least one cell cluster (6/21: M11, M12, M18, M19, M20 and M21) collectively represent 90.5%, 90.5% and 78.5% of all cells for *PICAFlow*, *FlowSOM* and *PhenoGraph* approaches, respectively. Furthermore, meta-clusters where *PICAFlow* effectively identifies at least one cell cluster (8/21: M7, M11, M12, M13, M18, M19, M20 and M21) represent 92.9% and 88.7% of cells identified by *FlowSOM* and *PhenoGraph* approaches, respectively. Together, these results show that the three methods tend to globally capture similar cell clusters if they are abundant enough. Unfortunately, the previously cited numbers also indicate that our method poorly captures very rare populations (typically < 1% of total abundance), which is the case for 10/21 meta-clusters: M1, M2, M3, M4, M5, M6, M14, M15, M16 and M17.

Noteworthily, these results also highlight the fact that our method works as intended, as it was basically designed to agglomerate clusters which share a very close phenotype, in the assumed purpose to reduce the number of final clusters to ease further interpretation and increase the number of cells per cluster and thus their respective statistical power. For instance, the *PICAFlow_C397* cluster seen in the M7 meta-cluster is the only one to be positive for the G6 marker when the *PICAFlow* approach is used. This M7 meta-cluster accounts for 3.7% of total cells with the *PICAFlow* clustering. This same G6^+^ population is instead split into 7 subpopulations for both *FlowSOM* and *PhenoGraph* methods, which inevitably leads to very lowly abundant subpopulations (0.02% only for *PhenoGraph_C21* and 0.14% for *FlowSOM_C9* clusters for instance). In our opinion, our method helps to dampen the generation of very lowly abundant clusters which could be unstable and/or present a low statistical power for further analyses. This observation is rather confirmed by the **Figure S1C** which clearly shows that our method effectively produces clusters which show an abundances distribution with a higher median (3.92%) and a narrower interquartile range (3.43) as compared to other methods (0.64% and 3.93 for *FlowSOM*, p-value = 0.048 as compared to the reference *PICAFlow* median, and 1.25% and 6.94 for *PhenoGraph*, respectively).

We also measured the percentage of positive cells for each marker (represented by red boxes around the clusters determined as positive for the marker of interest in **Figure S1B**) relative to the percentage obtained using manual gating (**Figure S1D** and **Figure S1E**). We observed that our method was the less derivative from 100% (mean percentage ± standard deviation of 98.46±24.58 for our method, 129.4±66.11 for *FlowSOM* and 149.2±131 for *PhenoGraph*) which represents the percentage obtained by the blind manual gating for each parameter independently (without any prior knowledge of co-expressed markers). Individually, *PICAFlow* is the closest to 100% for 10/13 markers, which is not the case for G6 (where it is ranked after *FlowSOM* but before *PhenoGraph*), Tbet (where it is ranked after *PhenoGraph* but before *FlowSOM*) and CXCR5 (where it is ranked after *FlowSOM* but before *PhenoGraph*). In a general manner, the conception of *PICAFlow* clustering method helps to avoid huge under or overestimation of each parameter overall signal. This is notably explained by the binary thresholds which are visually set by users during the last step of the clustering process.

In summary, our method tends to produce slightly less clusters than other methods, but is totally able to identify similar clusters as compared to other methods if they are abundant enough. On the contrary, our method does not identify well very rare populations (< 1%), but instead tries to merge them with their closest phenotypic neighbors, in order to reduce the number of final clusters and thus facilitate the subsequent interpretation of results. According to us, the produced clusters seem more reliable and statistically robust using our method. But, for the sake of compatibility and open-mindedness, *PICAFlow* still offers to users the possibility to alternatively use either *FlowSOM* or *PhenoGraph* clustering methods if they are unwilling to use our approach.

**Supplemental Figures**

**
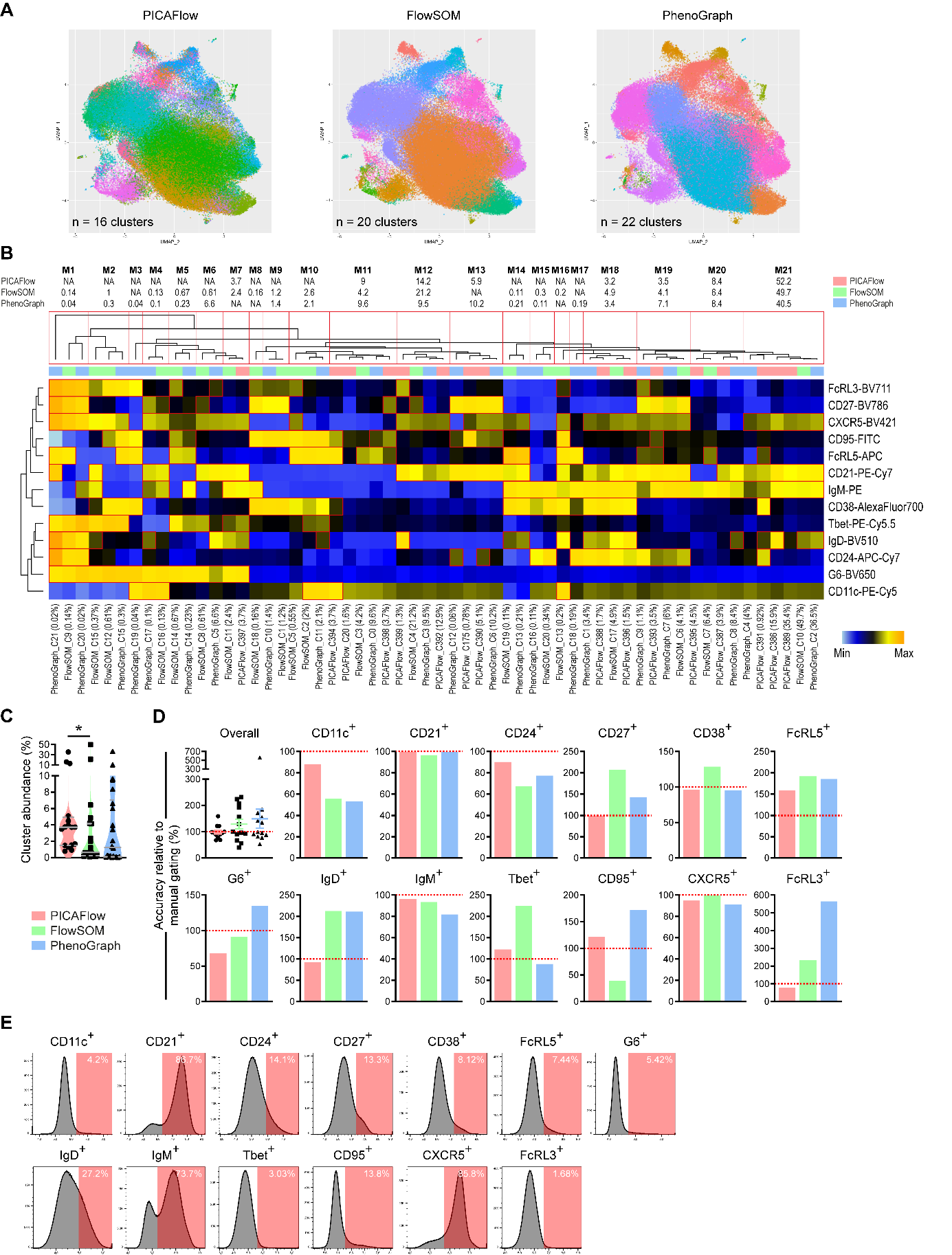
**

**Figure S1 – Comparison of *PICAFlow*-embedded cell clustering method with well-established *FlowSOM* and *PhenoGraph* ones.** The test dataset we provide in the tutorial was used to compare the clustering methods. First, lymphocyte-shaped single cells were gated and each channel was *logicle*-transformed using the dedicated R Shiny application. Then, compensations were adjusted and data were normalized before CD19^+^ CD3^-^ B cells were gated. Finally, the three clustering methods were independently applied to the same cells. **(A)** UMAP 2D plots showing the overlays of each cluster on the UMAP coordinates. **(B)** Heatmap showing the phenotype for each found cluster. Phenotypes were clustered using hierarchical clustering approach and colored according to the method that was used for its generation. Positivity of a cluster for a given marker is indicated with red boxes within the heatmap. **(C)** Scatter plot showing the overall cluster abundances obtained with each clustering method. Plain grey line represents the median and dashed grey lines represents Q1 and Q3. One-sample signed-rank Wilcoxon tests were used, considering each time *PICAFlow* as the reference. *: p-value < 0.05. **(D)** Scatter and bar plots showing the overall and individual accuracy for cell positivity identification. For the top-left scatter plot, colored lines represent the mean ± SEM. For each clustering method, positive cells for a given marker were the ones coming from clusters considered positive for this marker as shown by the red boxes in **(B)**. Accuracy is relative to the number of positive cells for each marker obtained by the manual gating shown in **(E)**. An accuracy of 100% means that the same number of positive cells was identified using both a given cell clustering algorithm and manual gating. NA = Not Available.
